# A Potential Protective Effect of Alcohol Consumption in Male Genital Lichen Sclerosus: A Case-Control Study

**DOI:** 10.1155/2023/7208312

**Published:** 2023-03-15

**Authors:** Joey El Khoury, Jessica Andraos, Anthony Kanbar, Rami Halabi, Serge Assaf, Anthony Mina, Sabine El Breidi, Charbel Dabal, Charbel El Hachem, Rodrigue Saad, Antoine Kassis, Maher Abdessater, Raghid El Khoury

**Affiliations:** ^1^Notre Dame des Secours University Hospital Center, Byblos, Lebanon; ^2^School of Medicine and Medical Sciences, Holy Spirit University of Kaslik, Jounieh, Lebanon

## Abstract

**Materials and Methods:**

A nested case-control study design was chosen. Subjects enrolled were adult male patients who had a circumcision between January 2010 and December 2020 at our university hospital, with a confirmed LSc diagnosis on pathology. Cases were matched with controls by age with a ratio of 1 : 1, all of whom were circumcised and had a negative pathology report. Data collection consisted of sociodemographic, behavioral, and past medical and familial history characteristics.

**Results:**

A total of 94 patients were enrolled. The mean age was 49.81 (±22.92) in the group of men with LSc. No significant differences in sociodemographic characteristics (age and BMI) were found between the two compared groups. Smoking cannot predict LSc as opposed to alcohol consumption, which we found to be a protective factor against the appearance of LSc (*p*=0.027). Men with LSc had significantly higher rates of diabetes (*p*=0.021) and hypertension (*p*=0.004). No associations were found between LSc and the presenting chief complaints, family history of LSc, and past penile trauma.

**Conclusion:**

In this study, we were able to compare multiple variables between 47 circumcised patients diagnosed with LSc and a control group. We found that LSc patients showed higher rates of diabetes and hypertension. A potential protective effect of alcohol consumption is to be explored in future projects with bigger sample sizes and higher statistical powers.

## 1. Introduction

Lichen sclerosus (LSc) is an acquired mucocutaneous disorder of unclear etiology with a predilection to the anogenital area. Even though it remains uncommon, extragenital manifestations exist and mainly concern the oral mucosa and the skin. In males, LSc primarily occurs in the genital area; anal involvements and extragenital manifestations are rarely found in this population [1]. Multiple nomenclatures have denoted LSc throughout the years, depending on emerging histological findings, clinical manifestations, or even lesion sites. Male genital LSc has been known as “balanitis xerotica obliterans” (BXO). This term is currently used for very advanced cases of LSc [2]. LSc affects both genders, with a female-to-male ratio varying from 10 : 1 to 5 : 1. The exact prevalence is unknown. The incidence ranges between 1/300 (0.33%) and 1/1000 (0.1%) in both genders [2].

The main clinical manifestation of LSc, dyspareunia, is the result of preputial and urethral dysfunctions. Although LSc can be silent and asymptomatic, it presents a wide array of sexual and urinary symptoms. The lesions can cause pruritis, tears, and sometimes bleeding. Phimosis, paraphimosis, and urethral strictures are some of the more common clinical signs. Subsequently, this dermatosis is considered to have a high burden on the patient's urinary and sexual well-being [3].

The diagnosis of LSc is mainly clinical, especially when the lesion is typical. A biopsy is seldom performed to confirm the diagnosis but is reserved only for suspicious cases of neoplasia, hyperpigmentation, or even for cases of clinical uncertainty [4]. Most often, these preputial lesions are described as atrophic plaques of ivory color or leukoderma, with sometimes the presence of hypertrophic scaly patches with telangiectasia and sparse purpura. In some cases, the glans can be eroded by the aforementioned lesions, especially in its premeatal area [5].

Topical corticosteroids have become the first-line therapy for LSc, having proven their efficacy. Symptoms are mostly relieved within the first few days of therapy. Other known alternatives and complementary therapies are moisturizers and immunosuppressors, such as topical calcineurin inhibitors, for patients who did not respond to the ultrapotent corticosteroid therapy or in whom the mainstay therapy is contraindicated [6]. In other cases, whenever medical therapy proves to be ineffective, surgical intervention is prompted. Circumcision is the main surgery performed, alongside other additional procedures when needed, such as a meatotomy or urethroplasty. Although surgery is considered highly curative, recurrences may rarely occur [7]. In addition, circumcision has been proven to play an essential role in improving LSc patients' quality of sexual life [8].

The etiologies and exact pathophysiology of LSc are still unknown, but what is known is that the connection between genital LSc and squamous cell carcinoma (SCC) is well established, and the prevalence has been estimated to be between 2% and 8% [9]. Throughout the literature, many assumptions were discussed but the subject is still debatable. Edmonds et al. and his associates published multiple studies about LSc, and suggested that genetic and environmental factors, inflammation, autoimmunity, and urine exposure play important roles in the pathogenesis of the condition; a genetically defined autoimmune pathway has been the most popular pathogenesis explored in the literature [3].

We aim, through this case-control study, to potentially associate genital LSc with risk factors and certain patient characteristics in Lebanese adult males.

## 2. Materials and Methods

### 2.1. Ethical Considerations

We started by obtaining the Notre Dame des Secours—University Hospital (NDS-UH) in Byblos, Lebanon's Institutional Review Board approval prior to data collection.

### 2.2. Study Design

We conducted a retrospective nested case-control study on patients who underwent circumcision in that establishment, between January 2010 and December 2020. All included cases were consenting male adults (>18 years of age) with a proven diagnosis of LSc on pathology.

### 2.3. Sample Selection and Matching

All cases of circumcision were screened for possible eligibility; 48 of those with a positive LSc diagnosis on pathology were selected. Only one case was excluded since the patient was deceased.

All cases are male; therefore, the matching process was based only on age, with up to a five-year margin. Besides age, no other factors were matched, and controls were selected randomly from patients who underwent circumcision with normal foreskin found on pathology during the same period (matching ratio 1 : 1).

### 2.4. Data Collection

Medical records for all patients were pulled, followed by data extraction of variables of interest, to minimize any recall bias. An excel sheet was filled with sociodemographic characteristics, past medical history (diabetes, hypertension, and others), habits, and allergies. We followed the data extraction process with a subsequent phone call to each subject to verify the accuracy and fill in any missing data. All 94 patients were compliant and consented to take part in this study. Alcohol consumption behavior was assessed using the “Alcohol Use Disorder Addiction Test” (AUDIT) which is a 10-item questionnaire with scores ranging from 0 for participants with low risk of addiction to 20+ for likely addicted patients [10].

### 2.5. Statistical and Data Analyses

SPSS v.28 (IBM SPSS Inc., Chicago, IL, USA) was used for the statistical analysis, with a 95% confidence interval (CI) and subsequently a 5% margin of error. We expressed quantitative values as averages and standard deviations, whereas qualitative values as frequencies and percentages. LSc associations with categorical variables were evaluated using the Pearson chi-square or Fisher's tests, depending on conditions. Other associations with quantitative variables were assessed using the independent samples *t*-test. Cramer's V was used to investigate the size of the effect of statistically significant associations. The level of statistical significance was set at *p* < 0.05.

## 3. Results

Control and LSc groups were compared searching for statistically significant differences, pertaining to sociodemographic, behavioral, and medical history variables, alongside the chief symptom on presentation (Table 1).

As planned, no significant age difference between both groups was noted (*p*=0.360). Furthermore, BMI, smoking status, the chief presenting symptom, the presence or absence of allergies, past penile trauma, or a family history of LSc were all found to yield no statistical difference.

Table 2 displays statistical associations between study participants and alcohol use, diabetes, and hypertension.

A *χ*^2^ test of independence was performed to examine the relationship between multiple sociodemographic and behavioral factors, the chief presenting symptom, and past medical history on one side, against having LSc. We have found that having diabetes is statistically associated with developing LSc (*χ*^2^ *=* 5.343, *df* *=* 1, and *p*=0.021) with an OR = 3.564.

Hypertension can also be added to the list of associated diseases in our sample. (*χ*^2^ *=* 8.428, *df* *=* 1, and *p*=0.004) with an OR = 3.938.

As for the magnitude of these associations, using Cramer's V as an effect size measurement [11], we found the previously mentioned statistical relations to be of medium (Cramer's V = 0.238) and strong (Cramer's V approximately 0.3) magnitudes, respectively.

Regarding alcohol use, we have found that low-to-moderate alcohol use was inversely associated with LSc (*χ*^2^ *=* 4.896, *df* *=* 1, and *p*=0.027). Having an OR of 0.365, alcohol use could potentially be a protective factor against LSc. That statistical relation is of medium strength (Cramer's V = 0.228) [11].

## 4. Discussion

Our research efforts focused on the clinical associations of LSc with medical and behavioral conditions. We have found higher rates of diabetes mellitus in men suffering from LSc, with an OR of 3.564 (1.165–10.903). This finding is well supported by the literature. Hofer et al. have found that diabetic patients were twice as likely to contract LSc in their sample of 485 men. Other associations found in that sample were a higher BMI and the presence of coronary artery disease in patients with LSc. This led Hofer and his team to support the hypothesis that lifestyle and metabolic variables may facilitate the development of LSc [12]. In another study conducted by Bromage et al., almost one third of diabetic subjects had a preputial phimosis [13], which was also the chief complaint of 23 patients in our sample inflicted with LSc (48.9%).

In 2015, another team of researchers was able to argue that hypertensive patients were twice as likely to develop LSc. In our sample, this odds ratio is increased to approximately four times. This study specifically links LSc to multiple metabolic syndrome components, including hypertension, an argument that does align with our findings [14]. Both of the aforementioned studies also highlighted that a higher than average BMI and smoking status were linked with LSc, contrary to our work where no significant associations were found [12, 14].

On the other hand, alcohol consumption was revealed to have a protective effect against LSc with an OR of 0.365 (0.147–0.904) in our sample. To date, and after an extensive search for published papers on that subject, no similar results were found. To explain this phenomenon, we are reminded that LSc is of unclear etiology and is rather multifactorial. Multiple papers have already postulated that the etiology of LSc is probably the irritable effects of urine [15, 16]. In our case, we would like to emphasize two suspected hypotheses: autoimmunity and vascular compromise of smaller terminal vessels [17].

The first hypothesis implies the presence of an autoimmune mechanism for the appearance of LSc lesions in the male genitalia. This pathway is poorly understood, especially in men, since female genital LSc is more cumbersome and thus more prone to better scientific research. The autoimmunity hypothesis arises from papers associating patients inflicted with LSc with autoimmune pathologies, highlighting a role for both cellular and humoral-mediated pathways. One of the earliest works on the matter was published in 1988, studying autoimmune disorders in 350 women with confirmed LSc on biopsy. They found that 42% of their sample had autoantibody levels at more than 1 : 20 and that 21.5% were diagnosed with at least one autoimmune-related disease [18]. Comparatively, a more recent paper examining vulvar LSc puts this prevalence at 28% and demonstrates the presence of elevated circulating autoantibody levels in their sample [19]. Contrarily, in an observational and descriptive case series of 329 patients with male genital LSc, only 7% of participants had autoimmune disorders, whilst less than 1% had a family history of LSc [3]. Other papers were able to associate LSc with localized scleroderma and morphea [20, 21] and autoimmune thyroid disease [22], concomitant with the presence of the respective positive serologic autoantibody titers. Furthermore, an in-depth look into the presumed immunoregulatory factors of LSc reveals a primordial role for CD4+, CD8+, and FOXP3+T-regulatory cells, as evidenced by stained immunohistology data from two different data sets [23, 24]. Taking into consideration all the aforementioned arguments, a postulated role for alcohol consumption becomes apparent. An interference of alcohol with LSc immunoregulation is of interest: impairment of CD4+ cell activation and proliferation by S-adenosylmethionine (SAMe) after catalysis by methionine adenosyltransferase II (MAT II). Ethanol, most commonly known as alcohol, reduces MAT II's enzymatic activity by reducing the transcription of MAT2A. In consequence, diminished intracellular SAMe levels lead to activation-induced caspase-3-dependent cell death (AICD) in T helper CD4+ lymphocytes [25]. Furthermore, other animal studies have found that alcohol abuse leads to decreased splenic cellularity and weight, and by consequence reduced numbers of CD8+ T cells. In alcohol-fed mice, CD8+ cell proliferation was proven to be reduced [26, 27]. Another theory was built around a similar rationale, discussing a potential beneficial role of alcohol consumption in oral lichen planus [28].

Second, metabolic syndrome may be incriminated in LSc, as supported by our data. Moreover, Hofer et al. were able to associate their sample of men with LSc with a higher BMI than their control group and increased rates of diabetes, a finding in common between both our investigations, in addition to increased rates of coronary artery disease and smoking [12]. Another published paper went to the extent of suggesting oral glucose tolerance tests and tight medical glycemic control as a way to improve cutaneous lesions in patients with diabetes mellitus [29]. Subsequently, a presumed compromise of genital microvessels is included in the multifactorial spectrum hypotheses of male genital LSc.

A protective effect of alcohol consumption on cardiovascular disease has been studied and proven on multiple occasions in academic papers. Moderate alcohol consumption is said to reduce the risk of diabetes mellitus type 2 by around 30% and lower the risk of mortality in diabetic patients [30]. In addition, moderate drinking was also linked to lower rates of morbidity and mortality in cardiovascular diseases, such as hypertension, peripheral artery disease, and strokes [31]. Another mechanism of action of alcohol on cardiovascular protection is via modulating inflammation. Alcohol reduces interleukin and C-reactive protein levels, resulting in a reduction of oxidative stress levels [32]. Vascular wall oxidative stress, more commonly known as the increase of free radicals fabrication at the expense of the physiological ability to counterbalance this phenomenon by the use of antioxidants, is essential in hypertension and vascular wall disease [33]. In a similar fashion, researchers have found that moderate alcohol consumption reduces all risks of microvascular complications in a cohort of diabetic patients [34].

Our study was limited by the sample size, which reflected the nature of the disease in question. LSc is a rare disease, thus the sample of only 47 affected patients was collected from our center. This is a thought-provoking paper, introducing new perspectives in understanding LSc on a global scale. A larger sample could achieve considerable conclusions regarding the role of alcohol consumption in LSc.

## 5. Conclusion

Our study aimed to compare multiple determinants between 47 circumcised patients diagnosed with LSc and a respective age-matched control group. We have found that in our sample, LSc was associated with increased rates of diabetes and hypertension. A potential protective effect of alcohol consumption was also found. Further research ought to be conducted on this subject to confirm this established connection between alcohol and LSc protections.

## Figures and Tables

**Table 1:** Sociodemographic and behavioral characteristics, medical history, and presenting symptoms of study participants (*N* = 94).

Variables	Controls *n* = 47	LSc *n* = 47	*p* values
Age (mean ± SD)	45.70 ± 20.25	49.81 ± 22.91	0.360
BMI (mean ± SD)	25.55 ± 3.57	26.00 ± 2.72	0.494
Presenting symptom			0.260
Phimosis	33 (70.2%)	23 (48.9%)	
Dyspareunia	3 (6.4%)	5 (10.6%)	
Recurrent infections	8 (17.0%)	10 (21.3%)	
Dysuria	3 (6.4%)	6 (12.8%)	
Accidental finding	0 (0.0%)	1 (2.1%)	
White lesions	0 (0.0%)	2 (4.3%)	
Smoking status			0.911
Nonsmoker	23 (48.9%)	21 (44.7%)	
Current smoker	17 (36.2%)	18 (38.3%)	
Previous smoker	7 (14.9%)	8 (17.0%)	
Alcohol use			**0.027**
No	10 (21.3%)	20 (42.6%)	
Low-moderate risk	37 (78.8%)	27 (57.4%)	
High-addicted	0 (0%)	0 (0%)	
Allergies			1.000
No	44 (93.6%)	45 (95.7%)	
Yes	3 (6.4%)	2 (4.3%)	
Diabetes			**0.021**
No	42 (89.4%)	33 (70.2%)	
Yes	5 (10.6%)	14 (29.8%)	
Hypertension			**0.004**
No	39 (83.0%)	26 (55.3%)	
Yes	8 (17.0%)	21 (44.7%)	
Penile trauma			0.495
No	47 (100.0%)	45 (95.7%)	
Yes	0 (0.0%)	2 (4.3%)	
Family history of LSc			0.435
No	45 (95.7%)	42 (89.4%)	
Yes	2 (4.3%)	5 (10.6%)	

BMI = body mass index. SD = standard deviation. LSc = lichen sclerosus. Significant *p* values of *p* < 0.05 are shown in bold.

**Table 2:** Inferential statistics comparing LSc determinants for study participants (*N* = 94).

Variables	Controls *n* = 47	LSc *n* = 47	*p* values	OR (95% CI)	Cramer's V
Alcohol use					
No	10 (21.3%)	20 (42.6%)	0.027	**0.365** (0.147–0.904)	**0.228**
Low-moderate	37 (78.8%)	27 (57.4%)			
Diabetes					
No	42 (89.4%)	33 (70.2%)	0.021	3.564 (1.165–10.903)	**0.238**
Yes	5 (10.6%)	14 (29.8%)			
Hypertension					
No	39 (83.0%)	26 (55.3%)	0.004	3.938 (1.517–10.218)	**0.299**
Yes	8 (17.0%)	21 (44.7%)			

LSc = lichen sclerosus. OR = odds ratio. 95% CI = 95 percent confidence interval. The bold values represent OR < 1, to highlight the protective effect, and Cramer's *V* < 0.3.

## Data Availability

The datasets used and/or analysed during the current study are available from the corresponding author upon reasonable request.

## Abbreviations

AUDIT: Alcohol Use Disorder Identification Test
BMI: Body mass index
BXO: Balanitis xerotica obliterans
CI: Confidence interval
HPV: Human papilloma virus
LSc: Lichen sclerosus
OR: Odds ratio
SCC: Squamous cell carcinoma
SD: Standard deviation.

## References

[1] Latini A., Cota C., Orsini D., Cristaudo A., Tedesco M. (2018). Male and female genital lichen sclerosus. Clinical and functional classification criteria. *Advances in Dermatology and Allergology*.

[2] Charlton O. A., Smith S. D. (2019). Balanitis xerotica obliterans: a review of diagnosis and management. *International Journal of Dermatology*.

[3] Edmonds E. V. J., Hunt S., Hawkins D., Dinneen M., Francis N., Bunker C. B. (2012). Clinical parameters in male genital lichen sclerosus: a case series of 329 patients. *Journal of the European Academy of Dermatology and Venereology*.

[4] Pérez-López F. R., Vieira-Baptista P. (2017). Lichen sclerosus in women: a review. *Climacteric*.

[5] Kirtschig G. (2016). Lichen sclerosus—presentation, diagnosis and management. *Dtsch Arztebl Int*.

[6] Fistarol S. K., Itin P. H. (2013). Diagnosis and treatment of lichen sclerosus. *American Journal of Clinical Dermatology*.

[7] Kulkarni S., Barbagli G., Kirpekar D., Mirri F., Lazzeri M. (2009). Lichen sclerosus of the male genitalia and urethra: surgical options and results in a multicenter international experience with 215 patients. *European Urology*.

[8] Czajkowski M., Czajkowska K., Zarańska K. (2021). Male circumcision due to phimosis as the procedure that is not only relieving clinical symptoms of phimosis but also improves the quality of sexual life. *Sexual Medicine*.

[9] Bhambhani D., Bhambhani S., Pandya N. K. (2022). Penile lichen sclerosis: a surgical perspective of its aetiology and treatment. *Cureus*.

[10] Babor T. F., Higgins-Biddle J. C., Saunders J. B. M. M. (2001). The alcohol use disorders identification test, guidelines for use in primary care. *Dep Ment Heal Subst Depend World Heal Organ*.

[11] Kim H. Y. (2017). Statistical notes for clinical researchers: Chi-squared test and Fisher’s exact test. *Restorative Dentistry and Endodontics*.

[12] Hofer M. D., Meeks J. J., Mehdiratta N., Granieri M. A., Cashy J., Gonzalez C. M. (2013). Lichen sclerosus in men is associated with elevated body mass index , diabetes mellitus coronary artery disease and smoking. *World Journal of Urology*.

[13] Bromage S. J., Crump A., Pearce I. (2007). Phimosis as a presenting feature of diabetes. *BJU International*.

[14] Erickson B. A., Elliott S. P., Myers J. B. (2016). Understanding the relationship between chronic systemic disease and lichen sclerosus urethral strictures. *The Journal of Urology*.

[15] Czajkowski M., Wierzbicki P., Kotulak-Chrząszcz A. (2022). The role of occlusion and micro-incontinence in the pathogenesis of penile lichen sclerosus: an observational study of pro-inflammatory cytokines’ gene expression. *International Urology and Nephrology*.

[16] Kravvas G., Muneer A., Watchorn R. E. (2022). Male genital lichen sclerosus, microincontinence and occlusion: mapping the disease across the prepuce. *Clinical and Experimental Dermatology*.

[17] Bunker C., Shim T. (2015). Male genital lichen sclerosus. *Indian Journal of Dermatology*.

[18] Thomas R. H. M., Ridley C. M., Mcgibbon D. H., Black M. M. (1988). Lichen sclerosus et atrophicus and autoimmunity—a study of 350 women. *British Journal of Dermatology*.

[19] Cooper S. M., Ali I., Baldo M., Wojnarowska F. (2008). The association of lichen sclerosus and erosive lichen planus of the vulva with autoimmune disease. *Archives of Dermatology*.

[20] Kreuter A., Wischnewski J., Terras S., Altmeyer P., Stücker M., Gambichler T. (2012). Coexistence of lichen sclerosus and morphea: a retrospective analysis of 472 patients with localized scleroderma from a German tertiary referral center. *Journal of the American Academy of Dermatology*.

[21] Lutz V. (2012). High frequency of genital lichen sclerosus in a prospective series of 76 patients with morphea. *Archives of Dermatology*.

[22] Aslanian F. M. N. P., Marques M. T. Q., Matos H. J. (2006). HLA markers in familial Lichen Sclerosus. *JDDG*.

[23] Terlou A., Santegoets L. A. M., van der Meijden W. I. (2012). An autoimmune phenotype in vulvar lichen sclerosus and lichen planus: a Th1 response and high levels of MicroRNA-155. *Journal of Investigative Dermatology*.

[24] Gambichler T., Belz D., Terras S., Kreuter A. (2013). Humoral and cell-mediated autoimmunity in lichen sclerosus. *British Journal of Dermatology*.

[25] Hote P. T., Sahoo R., Jani T. S. (2008). Ethanol inhibits methionine adenosyltransferase II activity and S-adenosylmethionine biosynthesis and enhances caspase-3-dependent cell death in T lymphocytes: relevance to alcohol-induced immunosuppression. *The Journal of Nutritional Biochemistry*.

[26] Song K., Coleman R. A., Zhu X. (2002). Chronic ethanol consumption by mice results in activated splenic T cells. *Journal of Leukocyte Biology*.

[27] Gurung P., Young B. M., Coleman R. A. (2008). Chronic ethanol induces inhibition of antigen-specific CD8+ but not CD4+ immunodominant T cell responses following Listeria monocytogenes inoculation. *Journal of Leukocyte Biology*.

[28] Xu X., Chen D., Mei L., Deng H. (2009). Is ethanol consumption beneficial for oral lichen planus?. *Medical Hypotheses*.

[29] García-Bravo B., Sánchez-Pedreño P., Rodríguez-Pichardo A., Camacho F. (1988). Lichen sclerosus et atrophicus. A study of 76 cases and their relation to diabetes. *Journal of the American Academy of Dermatology*.

[30] Koppes L. L. J., Dekker J. M., Hendriks H. F. J., Bouter L. M., Heine R. J. (2006). Meta-analysis of the relationship between alcohol consumption and coronary heart disease and mortality in type 2 diabetic patients. *Diabetologia*.

[31] Piano M. R. (2017). Alcohol’s effects on the cardiovascular system. *Alcohol Research: Current Reviews*.

[32] Ceni E., Mello T., Galli A. (2014). Pathogenesis of alcoholic liver disease: role of oxidative metabolism. *World Journal of Gastroenterology*.

[33] Ceron C. S., Marchi K. C., Muniz J. J., Tirapelli C. R. (2014). Vascular oxidative stress: a key factor in the development of hypertension associated with ethanol consumption. *Current Hypertension Reviews*.

[34] Beulens J. W. J., Kruidhof J. S., Grobbee D. E., Chaturvedi N., Fuller J. H., Soedamah-Muthu S. S. (2008). Alcohol consumption and risk of microvascular complications in type 1 diabetes patients: the EURODIAB Prospective Complications Study. *Diabetologia*.

